# A peripatetic pediatrician's journey into pediatric rheumatology: part III

**DOI:** 10.1186/1546-0096-5-17

**Published:** 2007-09-25

**Authors:** Earl J Brewer

**Affiliations:** 1 Houston, Texas, USA

## Abstract

Earl Brewer discusses his journey into pediatric rheumatology from 1958 to retirement in 1990 in three parts.

## Part III: the rest of the story

### VII Bureau Of Maternal & Child Health program to develop pediatric rheumatology 1980

The largest pivotal force that moved pediatric rheumatology forward was the decision by the MCH to bring pediatric rheumatology to the head of the list as a major unmet medical need in the care of children in the USA.

Even more important to pediatric rheumatology and to all of us caring for these children was the decision by the MCH to make pediatric rheumatology a model for implementing better care for all chronic illnesses of childhood. This included introduction of case management health teams. A national committee was formed to promote better understanding and availability of services for children with special needs in the educational system of our country. A valiant and slightly successful attempt was made to address insurance needs and deficits. For example, at this time, children with JRA were not only denied health insurance but even life insurance.

We were struggling to become an identified area of medicine. It was an uphill effort. We were the stepchildren of adult rheumatology, and the pediatric world did not know of our existence. There were few identified programs in medical schools. We had no depth in basic research. Care for children with arthritis was difficult for parents to find.

The BMCH was and still is a distinguished and modest federal agency that has as its main thrust the development of unmet medical needs of children. Few are aware that they developed the pediatric centers for hemophilia, pulmonary diseases, cardiology, and others.

Sometime in 1980 Drs. Merle McPherson, Director of Special Needs Children BMCH, and Dr. Vince Hutchens, Director of BMCH (then Division of Maternal and Child Health), contacted me and related that they had selected pediatric rheumatology as the next unmet medical need of children. It was as if the burden of the world had been lifted. They proposed that our clinic in Houston be a pilot project. Fifty thousand dollars was the allotted amount to get started. Vince Hutchens later told me that two other pediatric rheumatologists in our small world of pediatric rheumatology had cemented the deal with the DHHS and Congress. A pediatric rheumatologist contacted her senator from Washington State and another pediatric rheumatologist had contacted his senator from New York and complained that they should have been the pilot center. The Assistant Secretary called Vince and said that if there is that much interest in such a small area of medicine, the need must be there.

The first contract from 1981 to 1983 was directed through the Texas Department of Health from the BMCH in Washington. The premise was that if the contract were made with the state, then we would be more attentive to the wishes of the TDH. The premise was absolutely true. Dr. Punan Myer was the Chief of the Bureau of Crippled Children's Services. The idea of funneling our grant through the state was sound and promoted better success later.

The BMCH introduced the idea of team care and funding it. The team included a nurse educator, a physical therapist, an occupational therapist, and a social worker. Another concept was to establish outreach clinics in different parts of Texas. The first three outreach clinics were Port Arthur, Corpus Christi, and McAllen. Our team journeyed by car or plane for one day every other month. A local pediatrician was our liaison along with a base clinic. In Port Arthur, it was the Department of Health clinic in a poor section of town. In Corpus Christi, the clinic was held at the Driscoll Children's Hospital, and in McAllen, the clinic was initially held at the Lion's Club Clinic and later at a hospital.

The concept was that a local physician would follow the patients between visits by the team. Our health professionals also interacted with their counterparts. We thus not only gave expert care to patients and families but also gave professional education to health professionals in the town. The reception was great. Most of these and other clinics that we started in the early 1980s are still functioning today. We later extended the clinics to Providence Hospital in El Paso, Brackenridge Hospital in Austin, and Santa Rosa in San Antonio.

The BMCH staff received glowing reports about the concept and its reception. They had done this several times before for other chronic pediatric illnesses. This was almost old hat to them. It was an exhilarating experience for us. Each town was a challenge and required different skills to persuade the local medical community to participate. The Driscoll Hospital and Providence Hospital in El Paso were sufficiently persuaded that they each began centers for other chronic illnesses with specialists from out-of-town. I made many trips to both places before we had the clinics in place.

An interesting experience took place in Amarillo. Amarillo is in the panhandle of Texas. People from elsewhere don't focus that Texas is about 800 miles from north to south and about 800 miles east to west. It is therefore almost 800 miles from Houston to El Paso and from Houston to Amarillo. By air the trip to El Paso nonstop is two hours. From Houston to Mexico City the air flight time is only one hour and a half. The trip to Amarillo is a really long trip because it required at least one change of planes. They had two competing institutions as most small towns and cities did in Texas. I finally persuaded the two factions to sit with me, and we worked out a way for our team to come to Amarillo for our outreach program. The Chair of the Board of one institution said, " Doc, We folks up here are only a couple of generations away from cowboys and Indians fighting it out. We are very suspicious of the Fed offering us money to get started. My daddy was Chair and took what they called Hill Burton money to build the hospital and twenty years later they wanted the money back. We like your idea of the outreach clinic, but is it OK if we pay for it ourselves?" I told him it was OK and went back to Houston.

The McAllen outreach effort is an excellent example to tell in detail. We made first contact sometime in 1983. Dr. Joe McDonald was the local pediatrician who was interested in developing a pediatric rheumatology outreach clinic. He had a wonderful smile that filled the room. Children gravitated to him. His height was not great, and he had gentle hands as he examined children. They and everyone else felt at ease and trusted him. He was the ideal pediatrician. He and I bonded immediately and began our quest to persuade McAllen to accept our outreach concept. We persuaded the Lion's Club to house our bi-monthly visits with our team. We came by airplane and spent the night before the clinic. Joe's nurse was our local liaison. We later moved to the McAllen medical center for our clinics. When the grants were over, the effort here did not continue.

I learned a lesson about identical twins. Joe's identical twin brother was also a pediatrician in practice with him. Joe's wife told me that sometimes in the evenings the phone would ring, and Joe would say, "That's my brother and tell him, no." She would answer and sure enough, it was his brother. She would say, " Joe says, NO." His brother would say, "OK," and hang up. The ESP between them was real and a little scary.

The Providence Memorial Hospital in El Paso was a completely different situation to solve. The medical school was an embryonic branch of the University of Texas Medical School at Lubbock and was housed in the charity hospital. Providence was the largest and best hospital in the area at that time. The approach was to persuade the board of Providence to establish an outpatient facility for the area for chronic diseases in children. The administrator, Mr. Poteet, was a young, on-the-way-up guy who late became administrator of the larger Methodist Hospital of Lubbock, was the leading advocate of Providence Hospital's exercise of leadership in the community. Dr. Jorge Magana and Mandy Chew were also leaders in the effort. The clinic opened after about one year of planning and was a huge success. The clinic is still alive and successful in El Paso. Andrew Wilking, one of my fellows, has continued to fly to El Paso for the pediatric rheumatology clinic.

The Driscoll Hospital in Corpus Christi was successful due to Dr. Patrick Brosnan, a pediatric endocrinologist. This was the only outreach clinic established in a standing children's hospital. Organizational structure was different in each place. We had to adapt to the vagaries of the Driscoll Hospital. Our program of establishing liaisons with the local staff was successful only because of the dedicated efforts of Pat and his nursing staff.

The initial fifty thousand dollar pilot grant from the MCH in the early 1980s expanded during the remainder of the decade to several grants called appropriately, SPRANS grants or Special Projects of Regional and National Significance. The total approached a million dollars.

The central issue always was, is there life after the grant? Some institutions were able to make the transition and others were not. The program at TCH did survive after my retirement. The team program at the other institutions did not survive as I visualized they should, but then few projects do. The fact that team care survived at all made the effort worthwhile.

#### New Horizons in pediatric rheumatology – 1984

The three pilot center programs funded in 1981–2 were expanded to 6 centers by 1986. These centers were not the heart of the MCH program. A major conference in January 1984, "New Horizons for Pediatric Rheumatology" was sponsored by the BMCH in Houston. This conference provided a major forward thrust for our goals.

The meeting was held in Houston at the Houstonian. The participants were a who's-who of pediatric rheumatology in its infancy. The meeting was funded by BMCH. Merle McPherson and Vince Hutchins, our fairy godparents from the BMCH, were there, and we outlined our plans for expanding pediatric rheumatology. At this juncture we had several fellows in Houston. There was considerable skepticism about our efforts.

The program was a full-court-press effort. The Associate Commissioner for Health for Texas – Dr. Cliff Price, the Chief of Crippled Children's Services for Texas – Dr. Punan Myer, Dr. Fred McDuffie, Medical Director of the AF, and Dr. John Klippel from the NIH were participants. The program was comprehensive and laid out a plan for the development of pediatric rheumatology including team care and outreach clinics.

There were serious disagreements among our small, collegial group. Jerry Jacobs from New York City was troubled that I would travel to a small town like McAllen or Corpus Christi to give services instead of requiring the patients and families to travel to Houston. He considered the projects an inefficient use of professional time. I pointed out that many of these patients would never be seen because they did not have the money to travel to Houston. He felt that the money would be more efficiently spent to pay travel expenses for the families to come to Houston rather than the reverse.

Jack Miller from Stanford was a very conservative physician and also a caring and compassionate person. He was also one of our naysayers and was just as troubled as Jerry. He said, "Earl, we should not propose fellowship program expansion when you can't find jobs for your own fellows like Karyl Barron." (Karyl is now Deputy Director of the NIH Allergy and Infectious Diseases Institute in charge of intramural research.)

The meeting was a huge success, and the BMCH added several more centers creating enormous interest in pediatric rheumatology. Our center became a beacon for progress.

#### Family-centered, community-based, coordinated care for children with special needs [1]

The FCCCC project was a natural outgrowth of the BMCH relationship. In conjunction with development of pediatric rheumatology, Merle McPherson and Vince Hutchins were busy developing family-centered care with Bev Johnson, Dr. Phyllis McGrab of Georgetown Medical School, and Dr. C. Everett Koop, the Surgeon General of the US Public Health Service [Chick Koop].

My journey into FCCCC was in some ways fortuitous and in other ways destiny. Merle McPherson, Vince Hutchins, and Chick Koop expanded my horizon of proper care for children not only with arthritis but all chronic illnesses of childhood.

When the bonanza of financial help from the BMCH occurred in 1980–1, I felt elation that we would have funding to implement what we called our home treatment program of physical therapy and physician care. Merle and group had previously breathed life into such chronic disease areas as cardiology, cystic fibrosis, hemophilia, and other several others. Along the way they discovered that another unmet need was teaching physicians and other health professionals the lesson of family-centered care. Most doctors had a narrow view of their responsibilities and mission. Most pediatric care was crisis-intervention oriented. We were tuned to acute problems to be solved just as acutely with quick solutions. Physicians were like one of my colleagues who told that he had a partnership with all of his patients and families. He told them what to do, and they did it. He was serious.

I came to appreciate the basic tenet that families are the pivotal and central role in the care of children with chronic illness. When this concept is accepted, it naturally follows that families make the decision about care with support and advice from doctors and other providers. I learned that there were four basic tenets of community-based care: 1. Children with special health care needs need to live at home and to share in the every day life of the family. 2. Special school services are often needed with alternatives to classroom teaching with combinations of full-classroom teaching and combined part-time classroom and homebound teaching. Adaptive physical education, individual educational plans, and other facets must be considered and implemented. 3. Family-to-family networking as a community service to provide emotional support, teach families how the system works, and to guide families to accurate information is integral to FCCCC. 4. The last element is team care for the patient and family. 5, An overarching part is coordination of all of these services.

All of these concepts were implemented by various components of the several grants from the BMCH. The team of pediatric rheumatologist, nurse educator, social worker, occupational therapist, and physical therapist provided coordination and implementation of services. Toward the end of the grant cycle in the late 1980s, I persuaded Merle to allow us to hire Jeff Benjamin, a special educational teacher with the Houston Independent School District to be a member of the team and replace the social worker. This was one of the best moves we made. The teachers at the schools immediately accepted Jeff, as a special education teacher. He spoke their language and was able to implement special help for our kids. We had met a stonewall before Jeff's efforts. Later, after I retired, Jeff was made a school liaison for the entire Texas Children's Hospital clinic services.

Chick Koop, Vince Hutchins, and Merle McPherson called in early 1986 and asked if I would resign all of my activities at TCH, Baylor College of Medicine, and Kelsey-Seybold Clinic and work full-time on what became FCCCC. Ria and I had a ten-year plan every decade from 1970 onward. In 1980 our ten-year plan was to plan our goals around my retirement in 1990 to try my hand at writing nonfiction and fiction. The plan presented by Koop and colleagues was a whole new ball game in our eyes. We talked at great length and decided that both of us were excited about the idea of implementation of FCCCC. We decided to join the team. A decided advantage was that pediatric rheumatology would be used often to promote the concept.

Joining the team was an exciting experience for me. The professionals on board were the leaders of what became known as FCCCC. Phyllis Magrab was director of the Child Development Center at Georgetown University; John McQueen was past president of the AAP; Julie Beckett was mother of a child with severe respiratory disease and responsible for the Katy Beckett amendment where the government paid for home care of these children; Antoinette Eaton from Columbus, Ohio, was president of the AAP. The list was long and representative of the need of chronically ill children.

We started with a conference on October 16, 1986. From that meeting came the material for the Surgeon General's Report issued at our conference June 1987 by C. Everett Koop, MD. The meeting was a landmark meeting. Phyliis McGrab and I were co-chairs. We held it in Houston. It was a major event. Chick Koop put forth a seven-point plan aimed at coordinated care already discussed. Koop hoped that the forty-page report would motivate legislators and health professionals to adopt recommendations of the Campaign 87 Conference.

The report outlined a seven-point plan:

• Pledge a national commitment to FCCCC.

• Encourage community-based service systems.

• Assist in ensuring adequate preparation of providers of care.

• Develop coalitions to improve delivery of services.

• Establish guidelines to control costs of services.

• Encourage adequate financing.

• Promote research and education by using discretionary funds.

Dr. Koop outlined detailed goals under each point. The meeting was attended by a who's-who of pediatrics, as well as leaders from Washington and the State of Texas. The Commissioner of Health, Dr. Cliff Price, was a speaker. The AAP leadership was present and so many more. Senator Lloyd Bentsen, then chair of the Finance Committee of the Senate, was present and interested because his grandson had Down Syndrome. The program emphasized what we were doing in Texas. There was emphasis on the state level because we needed to promote FCCCC at each state level, and Texas was a good model to strive for success. The meeting and Report were good send-offs for our cause, but Chick Koop, Vince Hutchins, Merle McPherson, Dr. William Montgomery from the AAP, and possibly Dr. Richard Narkewicz, president of the AAP, met for a post-meeting. I was excited and euphoric about the new effort to be done. The AAP people were not as enthusiastic. The damper was a fear of the traditional physician losing control. There was also the cloud shading every aspect: How to persuade third parties to pay for FCCCC.

My assignment for this effort involved several thrusts:

• Promote and expand FCCCC including team care at more places in Texas and nationally.

• Persuade Blue Cross-Blue Shield of Texas to pay for FCCCC. Dr. Rogers Coleman, Medical Director of BS-BC was a friend and sympathetic to our goals.

• Develop a Family-To-Family Network in Houston and Texas

• Examine different ways to coordinate services effectively.

##### Promote and expand FCCCC including team care at more places in Texas and nationally

Pediatric rheumatology was the point chronic illness in these efforts.

The resultant publicity from the Surgeon-General's Report generated a lot of interest in our area as well as nationally. Dr. Howard Britton at Santa Rosa in San Antonio, Dr. George Edwards at the Brackenridge Hospital in Austin, Dr. Nina Harris in Bryan-College Station, Dr. John Pickett in Amarillo, and others expressed an interest in outreach, coordinated care programs. There was no federal funding for these programs, but George Edwards implemented team care at his hospital successfully.

State Senator Chet Brooks, then Dean of the Texas Senate, and Dr. Cliff Price, Associate Health Commissioner, engineered a statewide case manager program for chronically ill children. The numbers were always too few, and many times case management evolved into ways to deny service. We did a full-court press to persuade Senator Brooks and the Texas Senate to support case management. Kathy Angel, mother of Elizabeth Angel (a systemic JRA patient), our team, and myself journeyed to Austin on a hot day and went to the chambers of the Senate. Chet Brooks invited us to sit immediately in front of the committee. The lobbyists were restricted to the audience seats. This was important because even my own Texas Children's Hospital sent a faculty member to lobby against the case management legislation. One of the children told her story to Lieutenant-Governor Bill Hobby, the most powerful politician in our state. A Cyprus-Fairbanks school teacher refused to allow special help for her even though she could not hold the pencil in her hand because of her arthritis. The principal backed up the teacher. Governor Hobby took the child's hands, looked her in the eye, and asked, "Did your teacher really refuse to let you use something else?"

The little girl nodded. He asked, " What is her name?"

She told him and he said, "I'll call the principal and teacher this afternoon. Don't worry about help from now on."

The day was saved for the children. In addition, Senator Brooks added into the legislation a grant of $100,000 for us to start a Family-To-Family Network in Houston as a model to help special needs kids. This was the seed money which helped start F2F, now a highly successful parent's group.

##### Persuade Blue Cross-Blue Shield of Texas to pay for FCCCC

A key issue to address was payment for coordinated care. My thesis was that any medical or business activity that required more than five or six different people or entities to do a given job requires someone to coordinate the efforts for effective performance. This concept is economics 101 in the business world. The obvious need for a coordinator of services was not abundantly clear to insurance carriers such as Blue Cross, Blue Shield. BSBC was the original health insurance in the nation and started in Dallas in the 1930s. Rogers Coleman, a friend of mine and also a graduate of Baylor College of Medicine, was medical director of BSBC. We met in his Dallas headquarters. Rogers understood our goals and was sympathetic. He obtained permission to start a pilot project to show management that case management and coordination of services would be financially sound. We picked El Paso and the Providence Memorial Hospital. We already had approval for the specialty clinic there with Dr. Jorge Magana and Mandy Chew in place. The progressive administrator was Mr. Poteet. A team was hired. Rogers spoke at the Surgeon General's Conference. We were hopeful. When push-came-to-shove, however, the head of BCBS said, "Give me a double-blind study that shows that coordination of services is cost effective."

The reasoning, of course, was a circular equation with no solution. Insurance carriers never did buy into the program financially.

##### Develop a Family-To-Family Network in Houston and Texas

The Family-To-Family Network(F2F) began the night that I met Tina Bentsen Smith at the SG Conference and Report in June, 1987. Part of my thrust was to develop better visibility for our fledgling FCCCC. Senator Lloyd Bentsen graciously agreed to place his name with an award for a parent or professional making a contribution to FCCCC. The meeting was truly a national meeting, but my colleagues in Washington were concerned that I had stacked the deck with Texas participants. This was partially true. The auditorium was packed the night of the award ceremony, held at the Galleria Westin Hotel in Houston. I introduced Senator Bentsen, making one of my usual gaffes. He was an honored B-26 pilot in the Italian Theater in WWII. Instead of stating his Flying Cross Medal with four Bronze Stars, I said, "And Senator Bentsen was awarded the Flying Crock and four Bronze Stars." It enlivened the evening, and our friendship survived. I met Tina that evening, and the idea of F2F was formed.

When Senator Bentsen presented the first Lloyd Bentsen Award to Florene Poyadue from San Jose, California, for her contributions in developing Parents Helping Parents, all of my colleagues heaved a sigh of relief because finally someone not from Texas had won. When Florene came to the podium to receive her award, she said, "It is so great to be back in Texas again. I was a nurse in El Paso for seven years."

We organized F2F under the umbrella of the Kelsey Seybold Foundation and obtained a 501(c)3. Parents Helping Parents was our model. In rapid succession, we received a grant from the BMCH and $100,000 from the State of Texas to begin. Tina became the first chair. I made a serious mistake in hiring the first executive director, Pat Djuk, who was a marvelously qualified person. She had just finished her MPH at UT School of Public Health. Her master's thesis was compounding a list and short description of all of the special needs related entities in Harris County. Florene conducted several seminars to help us. She told me over and over again to hire a parent of a special needs child and ignore academic qualifications. She was correct. Pat was a good leader, but a parent was the only way to go for success. We also hired a wonderful social worker who was a man with no children. Again I failed to listen to Florene about the necessary early structure. Our social worker was leery about parents helping parents with support. The next executive director was a wonderful person from the school system, but again she did not have a child with special needs. Her brother had cerebral palsy. Unfortunately, the board we formed also never achieved fund raising status. The bottom line was that the F2F foundered.

In 1993 Tina and I met Eve Cugini, a mother whose child had cerebral palsy. She had worked as a manager in the retail business. She was on her way to a meeting of parents at a resort in Virginia. She was a breath of fresh air. Her enthusiasm, personality, effectiveness, and administrative abilities made her the heir to our foundering F2F. We turned over the 501(c)3, our records, and our support. F2F has been hugely successful since then because of Eve's leadership. She recruited other parents of special needs. Now, fourteen years later, F2F, which has had dreadful financial problems with its survival in peril every year, now is successful. F2F has several chapters, a statewide training program for parents paid for by the Texas Education Agency, and many more programs related to school special needs programs. F2F is highly respected by schools, state agencies, and local Houston foundations. This is all due to Eve Cugini and her dedicated volunteers. I have continued to be a member of the board and offer what help I can.

The cardinal tenets that form the core of F2F:

Give support to parents of special needs children. SUPPORT

Teach parents how to work the educational and medical system. EDUCATION

Direct parents to accurate medical and educational information. INFORMATION

##### Examine different ways to coordinate services effectively

This assignment was vague and evolved as I learned more from Merle, Phyllis, Bev, and Chick. They had the vision long before I appeared on the scene. I encountered incredibly blank stares from my colleagues who were section chiefs in the department of pediatrics at Baylor and service chiefs at Texas Children's Hospital. At one clinic chiefs' meeting, Dan McNamara, a mentor and friend, was a world-famous pediatric cardiologist. He looked at me and said, "Earl, if I sign on to your coordinated care team concept, do I have to call you for permission to use the team for one of my patients?" He was serious. During the Surgeon General's meeting announcing FCCCC, Dr. Koop and I conducted a press conference. He explained the concept well, I thought. The Channel 13-ABC reporter was a friend. She said, "Earl, it is a waste of time to give a long-winded intellectual discussion. You must bring a team with a child and parents, so your targeted audience can see and hear it." That was exactly how we had been successful with the Texas State Senate. We had to develop better ways to help our colleagues understand coordinated care. Chick explained it best in the preface to the report of our trip to Europe to examine chronic illness care systems in England, The Netherlands, Denmark, and Norway in 1987.

"This national agenda for children with special health care needs and their families was and remains a call to action to improve the care in health systems delivery so that services:

• Are focused on the families;

• Are provided as close to the child's home as possible;

• Are coordinated to ensure accessibility and responsiveness

• And are adequately financed."

**Figure 1:**
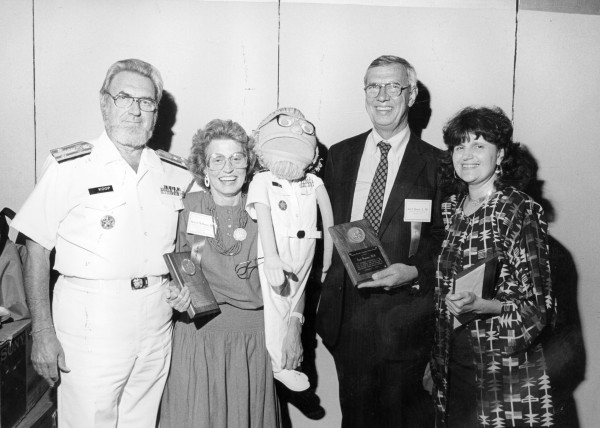
Surgeon General's Conference, 1988, Washington, DC. L R C. Everett Koop, Surgeon General, Merle McPherson, BMCH, Kids-On-The-Block puppet of Dr. Koop, Earl Brewer, and Phyllis Magrab, Georgetown University.

The imprimatur of the Surgeon General of the USA was a great door opener for our trip. The health authorities of the each country planned incredible trips to learn and see the best ideas and services. Our conclusions were published in Family-Centered, Community-Based Care: A European Perspective: Georgetown University Child Development Center Publication;1989. [2] [Figure 1]

Health policy: The most striking and important observation made by our group was the ability of the people in the Netherlands to join together in 1986 to forge a national health policy that continued the principles of the private insurance funding with a partnership with government yet maintained private control of the system. Funding was provided by insurance for virtually all people with only 16 percent contributed by the government. Selection by the patient of both the physician and hospital was maintained assuring freedom of choice by the individual. The nation prior to 1986 spent over 11 percent of its gross national product on health with yearly-uncontrolled growth. This was reduced to 8 percent. While the Netherlands is indeed a small country compared to the United States, both in size, ethnic and cultural diversity, and population, the principles still apply.

It is clear that we in the United States could profit from the Netherlands experience and models set forth by the Netherlands and work towards a similar goal to enable our country to assure not only basic health care but also exceptional expense health care for all of our citizens. We also can strive to better address deficient services, control costs more efficiently, and realistically consider the needs of patients rather than the perceived needs of providers.

##### Family Centered coordinated Services

In each of the countries visited, our group came away with a sense of how families were supported through the challenge of raising a child with special health care needs. In England, the home visitor program seemed to be a strong force in facilitating coordination and providing continuity of services, particularly for the infant and toddler age group. This program was felt by the medical leaders in England to be the single most unifying structure present. The !ionic Start program in England with parent befrienders seemed also to be a worthy program for consideration.

Of particular note in Norway is the Frambu Center that brings together families with specific special needs for one to two weeks in a camp setting for the purpose of parents meeting parents, children meeting children, health professionals reviewing the family's long term care plan and planning changes with the family. For a few, but not all groups it is possible to implement the new plan by sending a report to the community and by making telephone contact with various local health professionals to assure successful accomplishments of the task. Also the Norwegian concept of a contact nurse who is sent to the family's hometown to facilitate hospital discharge care plans is a concept that seems sound and should be evaluated in selected tertiary care centers in this country. Throughout our trip we were impressed by the concern for families and the variety of opportunities the countries had created. Public and private philanthropy often joined together to make things work. As in the United States, there is the increasing recognition of the role of the family in health care and the importance of parent-professional partnerships.

A number of projects came from this trip. The Frambu camp in Norway blew me away with its simplicity and effectiveness for a small country with a large rural population. The camp was sequestered in the hills near Oslo and was idyllic. Children with a serious chronic illness and their siblings and parents went to Frambu with other families for a week. A different chronic illness was covered each week. Specialists and appropriate health professionals also came. There were probably five or more families at the camp. The professional examined the child and family to assess what medical, school, and social needs were needed. The family and team then agreed on a treatment plan. There was a lot of interchange among the families and staff. After leaving the camp, a nurse practitioner went with the family to their hometown and met with the local physician, school, physical therapy, and whatever needs there were. She followed up in a month or so. They had excellent data to show how much improvement occurred with this form of FCCCC.

I was able to interest a group in Salt Lake City who cared for children with chronic respiratory illness in this concept. They instituted such a camp in the Wasatch Mountains. The last contact I had with them, the camp was continuing. The outcome of this kind of effort was that more and more pediatric chronic illness centers established camps for kids but not their parents. Pediatric rheumatologists led the way with these camps for several reasons. The pediatric rheumatology team could go to the camp and provide expertise and fun. Regular camps would not accept children with special needs because of worries about liability and special equipment that might be necessary.

A second project patterned after the Frambu camp was started by Jim Bomar and his wife. Jim was a prominent attorney and politician in Texas and lived in Temple. He was a deeply religious man and dedicated 1200 acres or so to the Peaceable Kingdom Retreat. We arranged with the Scott and White Clinic in Temple and with the Texas A&M medical group to send children to the camp. We later tried to enlist clinics in Austin and San Antonio. Success was spotty. The turf problems and lack of understanding of FCCCC were incredible. Team coordinated care was so clear to me, but not to others. Paying for it always dealt a fatal blow. The camp is now run by his daughter and son and seems to be flourishing.

In Houston a Camp-For-All was done by dedicated parents and physicians at the Texas Children's Hospital and is flourishing, as are camps all over the country.

### VIII Rheumatology Section AAP 1981

Dr. Gerald Hughes was executive director of the AAP in the late 1970s. Dr. Betty Lowe, Professor of Pediatrics, at the University of Arkansas, was on the board of the AAP and interested in pediatric rheumatology. She spent time with us in Houston in the early 1970s. Betty had been a resident at the Children's hospital in Boston a few years after I was there. Both Jerry and Betty were supportive of establishing a rheumatology section of the Academy. Both were essential in shepherding the application to acceptance. The first meeting of the new section was on the occasion of the fiftieth anniversary of the AAP in Detroit, Michigan in 1980.

Interested members of the Academy held an election for the membership of the section committee. The original committee members were Balu Athrea, Jack Bass, Virgil Hanson, Jerry Jacobs, and Carol Lindsley and myself. An election was held for the first chair. I was elected chair.

We were successful in our quest with the AAP. Everyone wanted to increase the knowledge of pediatricians in general, as well as provide a home to the developing field of pediatric rheumatology. We were included prominently in the programs of the Academy including the annual, spring, and seminar meetings at different locations in the country.

I was elected to be secretary of the Sections Committee and a member of the Drug Committee of the Academy. Thus, we made a major breakthrough of acceptance for pediatric rheumatology. This breakthrough came with a double-edged sword. Within a year I was summoned to the Board of Directors of the American Rheumatism Association to explain why we were breaking away from the ARA. I explained that we were simply expanding our horizons to increase knowledge of general pediatricians. Jerry Rodman of Pittsburgh, a legend of rheumatology, looked at me with a woeful expression and lamented that we were the lost sheep and needed to come home. Dan McCarty wrote an editorial in A & R journal and lamented that the prepubertal panthers had jumped the corral fence and should come home. A few years later, Dan and I spoke at the annual UCLA seminar at the Annenberg Center in Palm Springs. I prepared slides of prepubertal panthers jumping back into the corral.

The AAP Rheumatology Section has survived and sponsored two Park City meetings. The membership has not flourished as one might hope, but both the ACR pediatric council and the AAP Rheumatology Section work together with a collegial spirit.

I feel honored that Academy created the annual Earl Brewer Travel Award to honor a pediatric rheumatology fellow for outstanding research. It is so important to promote the younger physicians coming along in our field. The initial recipients all have successful academic careers.

### IX AJAO 1980

The American Juvenile Arthritis Organization of the Arthritis Foundation (AJAO) held its first meeting in 1981. The initial group: Arlene Johnson, president, Ohio; Richard Weaver, vice president, California; Janna Zeltwanger, secretary, Indiana; members: Barbara Barrett, Washington; Jim Cassidy, Dawn Hafeli, Michigan; Joseph Levinson, Art Choate, Ohio; and Al Moske, Virginia.

The AF was not supportive during these times, but the parents wanted the needs of children and their families to be heard. An immediate project was the proposed Juvenile Arthritis Centers to be sponsored by the AF. I had spoken to this issue at the AF Government Affairs Committee.

The group organized itself with appropriate committees and by-laws. Kathy Angel, a Houston parent of Elizabeth who had systemic JRA, and I met with the board in 1983 and 1984. We proposed a national meeting and visited the mountain resort of Keystone, Colorado, as a possible site. The national AF staff believed that we could not possibly pull off a meeting by the summer of 1984 in Keystone. Kathy was a singleminded mother with a cause. She was also able, effective, and smart. Kathy created a command center in Houston and engineered video coverage, special help for the children, and handled the myriad of details necessary for such a meeting.

The big week came, and it was truly a landmark meeting for the cause of children with arthritis. The children were elated and had a great time. They met other children with arthritis for the first time in many cases. One child from Australia came in a wheel chair. A few years later he came to an AJAO meeting walking. We had several days of talks and seminars. The meeting was indeed an epiphany.

From this seminal meeting, the AJAO found its place in the heart of the Arthritis Foundation and pediatric rheumatology. Today it remains an even more important part of the AF. I was pleased when the annual Earl Brewer Award was created in 1989 to honor outstanding achievement by a health professional.

### X Pediatric Rheumatology Board 1980–1990

I formerly applied to the American Board of Pediatrics in 1980 to ask them to create a sub-board of pediatric rheumatology. The application created a resounding thud. The lack of response was monumental. There was no reply essentially. Several colleagues, in particular Jane Schaller and Jack Miller, felt that there was not a sufficient body of knowledge to justify a board. Immunologists wanted to claim the children with arthritis. In the mid-1980s, Jim Cassidy, Patience White, and Deborah Kredich mounted a massive effort to gain acceptance of the application. Finally in 1990, Dr. Robert Brownlee, President of the American Board of Pediatrics, and I went to the annual meeting of the American Board of Medical Specialties for approval of pediatric rheumatology as a sub-board. Dr. Brownlee and I waited with great trepidation for our turn with the board. We listened with sinking hearts as the board members skewered the preceding applicants for emergency medicine and intensive care. Their applications were tabled for that year.

When Dr. Brownlee and I came to the table before the board, we braced ourselves for an inquisition. We had practiced responses of course, but we were nervous. The chair greeted us with a smile and said, "Welcome." That was a good start. He then told us that the board had carefully studied the written material, and they were pleased with the application. We were breathing a little easier but were waiting for the other shoe to drop.

The chairman said, " Welcome to the ABMS group of approved subspecialties." This was the last item on my list of projects to complete. My journey in pediatric rheumatology was over.

### XI Postdoctoral training programs

It seems appropriate to discuss postdoctoral training at the end of my journey. At the beginning of our quest to develop pediatric rheumatology, the thought of replicating our knowledge and training others was not an item of immediate concern. We spent time with adult rheumatologists, and indeed, several members were adult rheumatologists. The members of the initial collegial group were basically clinicians with a smattering of laboratory skills and training. Eventually, more basic research skills were required as well as more clinical research skills. Observational and scientific clinical research was necessary at the beginning from the 1950s to the middle 1970s. Toward the end of the 1970s, programs began to add fellows for training.

Funding for our program began as a trade-off bargain with the administrator of the hospital, Newell France. The year was 1976. I was asked to be the president of the medical staff at Texas Children's Hospital. All of the many programs already discussed for pediatric rheumatology were bouncing in the air at the same time. The last thing that I needed was to be president of the staff for a year – in particular the coming year, 1977–78. Dr. Blattner, my chairman and mentor, was retiring. He and I had shared office space for fourteen years. A new chairman of pediatrics would be selected. The Denver children's Hospital had almost disintegrated with their change-of-command a year or so earlier. I represented so many facets of the hospital and practicing medical community that Newell and the staff felt that I could maneuver the changing-of-the-guard better. I still did not want to do it at the possible expense of other projects. Then Newell hit me where I was totally vulnerable. He said that if I would be president, he would fund two pediatric rheumatology fellows per year – An offer I could not refuse. The year of retirement and changing-of-the-guard was indeed tumultuous but successful. Dr. Blattner retired and Dr. Ralph Feigin was selected as the new Chair and Physician-in-Chief. To his credit, he never reneged on the deal, even though he didn't make it.

Rob Nickeson was the first fellow added at TCH/Baylor. Rob was a graduate of Yale University and Pittsburgh Medical School with pediatric training at Pittsburgh. He was incredibly bright. His boundless and irresistible energy were apparent when he entered the room. Rob did not just walk into the room; he burst into the room. His reddish hair and beard, darting eyes behind rimmed glasses, and his breathless, " What's going on?" let us know that Rob was here. His speech was rapid and clipped. He was as close to hyperactivity as one could be. He sang in an acappella choir at a Methodist Church and worked with disadvantaged youth. We were able to channel his incredible energy into seven research projects while he was a fellow along with his clinical duties. He went to the University of Oklahoma as the first pediatric rheumatologist for several years and started an outreach clinic in Tulsa. Later he and his wife, Nadine, moved to Florida. He is now in charge of the pediatric rheumatology program at the University of South Florida.

Karyl Barron was equally bright, a Phi Beta Kappa at the University of Miami and an AOA at Emory Medical School. She did her pediatrics residency at Emory. In the same way that Rob was hyperactive physically, Karyl was hyperactive mentally. It's always challenging to direct someone brighter than you are, but I tried. Karyl absorbed knowledge like a sponge and quickly mastered clinical material and learned new laboratory research skills at the same time. She later spent time in Allergy and Immunology. Karyl, married with children, now is Deputy Director of NIAID (National Institutes Allergy and Infectious Diseases) in Bethesda. She is a leader and a great pediatric rheumatologist.

Andrew Wilking was a graduate of Harvard College and Columbia Medical School. He did his pediatric training at Columbia. He was at least six feet five inches tall and skinny as a beanpole. His curly hair flopped over his forehead, and the twinkle in his eye behind large glasses and a wonderfully reserved smile made him like a favorite next-door-neighbor. He was married to Marilyn, an AOA at Columbia and a leader of the outpatient department. She came to Houston willingly and worked as a pediatrician in Dr. Frank Hill's pediatric group. They were a great couple. Andrew's mother was a prominent pediatric psychoanalyst in New York City, and his father was chief-of-staff at St. Luke's Hospital in New York City.

Andrew and I went head-to-head soon after he arrived. He had a very good idea that we should have Saturday morning teaching rounds for the entire morning. The idea was wonderful, but that is not how we structured the time schedule of the program. In addition, I asked each fellow to select several research projects to perform during fellowship. We worked together to select the projects. Andrew told me that he was only going to teach and take care of patients in his career. We were at a stand off during his entire fellowship.

Andrew has done precisely that in his successful career. He has been honored for his teaching skills at Baylor where he remains on the faculty. There is even a master's degree program designed by him that is offered by Baylor. He also bought into the idea of outreach clinics and continues to travel to several cities and towns in Texas. We are now good friends.

Daniel Lovell waited a year working in an emergency room in Kansas City before we had an opening for a fellowship. Dan was a low-key, extremely bright doctor with a droll sense of humor and a workaholic temperament. He was slight-of-build with reddish hair and beard. He and his wife, Anne, hated Houston from the time they arrived until they left. They came because Dan liked our program. Ed, Dan, and I bonded immediately and remain in touch to this day. After Dan came for his interview, it is a wonder that he accepted our offer. I took him to a contentious meeting of the local AF chapter where an adult rheumatologist explained to me that I should tell Judge Hofheinz, owner of the baseball team, the Astrodome, and Astroworld, that his kind offer of a benefit for our pediatric program should be refused and held instead for the adult arthritis program. I explained to the adult rheumatologist that his request was not going to happen. Dan and I had several old-fashioneds to provide the restraint necessary.

Dan conducted several research projects, which were duly published. In addition, he completed his MPH at the University of Texas School of Public Health. Dan went to Cincinnati from Houston, and I asked the PRCSG to elect him chair on the occasion of my retirement in 1990. He and Ed have taken the PRCSG to heights I never imagined. He is now Professor of Pediatrics and the Joseph E. Levinson Chair of Pediatric Rheumatology, as well as chair of PRCSG and a host of other successful activities.

Mary Moore was from the Pennsylvania area and was a shy, low-key, warm, friendly, quiet lady. Her large eyes focused intently on you, and she listened carefully to what you said. Mary was very bright and was an observer of life. She was an excellent physician. When she gave an important research paper on heart block in neonatal lupus to a combined group of neonatologists, cardiologists, and rheumatologists at TCH she explained to the group that she felt at ease sitting rather than standing at the podium.

Mary had a practical Midwesterner's approach to life. One time I remonstrated with an older patient who had graduated from college and still lived at home. I thought that young people should begin their new adventures in life in their own digs. Mary said, "In my upbringing girls stay at home until they are married." Carmen De Cunto chimed in with an equal blast. I was more careful after that about inserting my own bias.

Mary moved to the University of Iowa and is now in Michigan in a clinic at Michigan State Medical School.

Xiaohu He provided my first opportunity to pick a fellow from overseas. What an adventure! Professor Jiang Zaifang, Director of the Research Institute in Beijing, China, spent a year (1982) at TCH observing and noting what skills and knowledge would be useful to China. She decided that pediatric rheumatology was one of the unmet needs. There is a great deal of lupus in China. In 1983 I received an invitation to be a guest of the People's Republic for three weeks; furthermore I could bring a group with me. The ostensible purpose was a medical cultural exchange. The real reason was for Professor Ziang and me to pick one of her pediatric faculty to come to Houston to train in pediatric rheumatology. Fourteen people went, including Ria. Our adventures are worthy of an entire article. The trip expanded my horizons beyond all belief. In brief, we gave a one-day seminar at the Beijing Children's Hospital. We were then given a two-week tour of the country in such places as Xian, Hangzhou, Shanghai, Guilin, Hong Kong, and other cities. Professor Ziang and I selected a senior pediatric nephrologist, Dr. Xiaohu He to come to Houston for three years and train as a pediatric rheumatologist.

Xiaohu was in her forties, extremely bright, and effective. She was small in stature and slim. Her ready smile and hard work endeared her to everyone. She had a ten-year-old son. Her husband was an executive in the mining industry. Typical of the Communist system of that era, her husband and son were required to stay in Beijing for the three years. It was very hard on Xiaohu. She worked successfully on several clinical research projects with Ed Giannini and others in our group. When Xiaohu came to Houston, I found quarters for her at Favro Hall, the nurses' dorm in the Texas Medical Center. It was only a block or less from TCH. She ate in the nurses' cafeteria. Within a few months her weight was noticeable increased. In Beijing she had bicycled 11 miles daily to and from work. The starchy diet of the dorm was the culprit. She was a leader in the Chinese scholar community in Houston. There were many scholars at the time, and the Chinese consul always asked her to mediate problems with the other scholars. I only had funding for two fellows from the hospital. I turned to my friend Fox Benton, a highly successful oilman, for help. We went to Ambassador Kenneth Franzeim for his help for one year. Foxy paid for the other two years.

When Xiaohu returned to Beijing, she developed the cause of pediatric rheumatology beyond my wildest dreams. She established at least 15 pediatric rheumatology clinics in China. Xiaohu was recently President of the Chinese Pediatric Society. I hear from her yearly and hope to visit her again.

Carmen De Cunto provided my next foray into foreign fellows. My plan was to take fellows who had a definite place to which they would return when finished with their fellowship. A physician in Zurich referred Antonio Aranda. Antonio and his family lived in Buenos Aires. Antonio had severe polyarticular JRA and wheelchair-bound with severe and constant pain. We admitted him to the hospital for two weeks or so. Our program already discussed was amazingly helpful. He came to the hospital in a wheel chair and walked out. His parents, Jose and Mimi Aranda, and I became fast friends to this day. They gave much of the funds spent on the first AJAO meeting in Keystone, Colorado.

Jose was the publisher of the largest Spanish language newspaper in the world, The Clarin, in Buenos Aires. The Arandas came to Houston monthly for several years. Jose decided that Buenos Aires needed a pediatric rheumatologist. So, Ria and I visited there several times over the next several months and selected Dr. Carmen De Cunto to come to Houston for pediatric rheumatology training for three years. She was married to a nice young man who was an engineer and spoke no English. We arranged for him to pursue a master's degree at the University of Houston. The Arandas paid Carmen's expenses and salary. After finishing her training, she returned to Buenos Aires to the Italiano Hospital and has become a leading pediatric rheumatologist in South America and Argentina.

Carmen was interested in streptococcal disease and post streptococcal reactive arthritis and wrote more than one paper with the cardiology section at TCH.

Abraham Gedalia came to us from Be'er Sheva, Israel. He spent six months with John Baum at Rochester, New York before joining us for two years. Avi was older than the younger fellows and had been in practice in Be'er Sheva for several years. He was one of best clinicians whom I have known. He also was soft-spoken and soft-mannered. His face could have been a Norman Rockwell portrait. His visage spoke compassion and kindness. With his deeply lined, olive-complexioned face, he could calm the most upset child with a soothing hand and voice. Avi focused on hypermobility and arthritis in children and did an epidemiologic study at the Awty private school. This study and other papers were published later.

Avi was married to Fania, who had emigrated with her parents from Estonia as a teenager. She had a scholarship in Rega after high school. Only about one or two percent of the old Soviet-era students won such awards. Her parents emigrated to Israel to escape Russian oppression, and she ended up pulling onions on a commune farm in Israel. She said to her mother, "I left a scholarship in Rega to pull onions on a commune in Israel. This is the free world?" Life did become better, and she became a public health nurse. She and Avi met and married.

Avi returned to Israel after his fellowship for a few years. He was such a great pediatric rheumatologist that we simply could not let him stay in Israel. Several of us worked the system and Avi became a professor at LSU in New Orleans at the Children's Hospital where he is today in 2007.

Ivonne Arroya burst on our scene from Puerto Rico. She arrived for an interview and disrupted the entire office with each step as she walked into the section. She truly looked like a South American movie star and was dressed for the part. She also was personable and smart. She was a breath of fresh air. Her English was perfect but heavily laced with accent. She was primarily interested in clinical skills and worked with Avi Gedalia in the hypermobility studies. Ivonne returned to the University of Puerto Rico and is a successful pediatric rheumatologist in San Juan.

## Overview of changes in care and knowledge 60s, 70s, 80s, 90

At the end of the 1950s and early 1960s bed rest, aspirin, and some use of corticosteroids were about the limit of care. The other NSAIDs were added to our armamentarium, and better pain relief and reduction of joint swelling improved the lot of children. The more effective relief of pain aided in active exercise programs and increased activity. The concept of integrating the arthritic child into the family spectrum and treating him/her as a normal child moved forward.

We joined forces with Dr. Sidney Cleveland, a psychologist, to promote and learn more about body image and working with families as a group. We began to pay attention to persuading the schools to integrate the children into as regular a routine as possible in the schoolroom. We promoted the idea that the family is in charge of the care of the child, and the physician and health professionals are advisors to them. We learned to include the families in care and care decisions better.

Slower acting medicines came into widespread use. Methotrexate for a long time was the best. Then the medicines that blocked inflammatory mechanisms in the body provided even more help.

We teamed early with Dr. Malcolm Granberry, an orthopedist, to learn better ways to improve function of the muscles and joints. We evaluated synovectomies, tendon lengthening, and joint replacement. We learned that each has a place. We even looked at acupuncture with Dr. Xiaohu He while she was here from China.

Underlying all of the improvements were the incredible advances in our knowledge of the genetics and immunology of rheumatic diseases in general and children in particular. My personal participation in these endeavors was limited, and the story will have to be told by others.

In the 1950s many children were in bed or wheel chairs. By the 1990s, few children were confined to wheel chairs or crutches. Indeed it has been a satisfying journey. There is much to be done, but there is an able group of young people better equipped than I to carry the challenge into the future.

## Abbreviations

AAP American Academy of Pediatrics

ACR American College of Rheumatology

AJAO American Juvenile Arthritis Organization

TCH Texas Children's Hospital

AF Arthritis Foundation

FCCCC Family-Centered, Community-Based, Coordinated Care

## Competing interests

The author(s) declare that they have no competing interests.

## References

[B1] Brewer EJ, McPherson M, Magrab PR, Hutchins VL (1989). Family-Centered, Community-Based, Coordinated Care for Children with Special Health Care Needs. Pediatrics.

[B2] Magrab PR, Brewer EJ, McPherson M, (with introduction by Surgeon General CE Koop) (1989). Family-Centered, Community-Based Care: A European Perspective.

